# Advantages of asynchronous online focus groups and face-to-face focus groups as perceived by child, adolescent and adult participants: a survey study

**DOI:** 10.1186/1756-0500-7-756

**Published:** 2014-10-24

**Authors:** Marieke Zwaanswijk, Sandra van Dulmen

**Affiliations:** NIVEL, Netherlands Institute for Health Services Research, P.O. Box 1568, 3500 BN Utrecht, the Netherlands; Department of Primary and Community Care, Radboud University Medical Center, Nijmegen, the Netherlands; Department of Health Sciences, Buskerud and Vestfold University College, Drammen, Norway

**Keywords:** Online focus groups, Survey, Face-to-face focus groups, Advantages, Perceptions

## Abstract

**Background:**

Online focus groups (OFGs) are increasingly used as a method of data collection. Although their advantages for research have repeatedly been described, participants’ opinions about OFGs have seldom been studied. We investigated OFG participants’ preference for participation in an OFG or a face-to-face focus group (FTF), as well as their perceptions of the advantages of both methods. We also investigated whether any differences exist between the perceptions of child, adolescent, and adult participants.

**Methods:**

Participants’ opinions were studied by means of a questionnaire completed by 284 persons (aged 8–72 years) after their participation in one of 50 OFGs. The OFGs were conducted between December 2005 and December 2013 as part of 19 separate studies. Chi square tests with p <0.05 were used to test differences in perceived advantages of OFGs and FTFs between children, adolescents and adults.

**Results:**

The most important advantage of OFGs as perceived by OFG participants was the possibility to participate at a moment most convenient to them. Adolescents and adults (90.5% and 95.9%) more often reported this as an advantage than children did (30.8%, p < 0.02). Another important perceived advantage of OFGs was the possibility to participate from home (69.1%). The most important advantage of FTFs was respondents’ perception that it is easier to have a discussion with the whole group when there is personal contact with others (48.5%). This advantage was mentioned significantly more often by adults (78.4%) than by children and adolescents (4.8% and 17.7%, p < 0.02).

**Conclusions:**

Participants’ perceptions of OFGs partly concur with the advantages of OFGs as a research method. Whereas respondents generally value the convenience of participating at their own time and place, the anonymity of OFGs and the increased ease to discuss personal issues were mentioned less often as advantages by the participants. An aspect that may need more attention when conducting an OFG, is the absence of a fluid discussion, which is, according to our respondents, easier to achieve in an FTF. This underlines the importance of the moderator in enabling a constructive discussion.

## Background

The Internet is increasingly being used as a medium for collecting data for scientific research [1–4]. Online focus groups, also referred to as Internet-based focus groups, electronic focus groups, chat-based focus groups, or virtual panel discussions, are one of these Internet-based research methods. The feasibility and effectiveness of online focus groups (OFGs) have been clearly demonstrated for various age groups [2, 5–9], although a recent study reported less favourable results for young children [10]. OFGs have several advantages compared to traditional face-to-face focus groups (FTFs), for participants as well as researchers [2, 6, 7, 11–13], while producing similar amounts and quality of information [14]. Firstly, they allow spatiotemporally separated participants to join the discussion from their home and at a convenient time. Secondly, the higher level of anonymity in online discussions is perceived to allow participants to speak more freely and provide more honest answers, particularly regarding sensitive topics [13, 15–17]. Thirdly, the written contributions of participants yield immediately available data, which considerably decreases costs and time needed for data entry and analysis.

Studies until now have mainly focused on the advantages and disadvantages of OFGs for researchers and the quality of the produced data. Participants’ opinions about the advantages and disadvantages of OFGs have, however, seldom been investigated. An exception is the study by Reid and Reid [16], who found that participants’ main reasons for preferring participation in an OFG instead of an FTF were: (a) the greater amount of time to think about their feelings in an OFG, (b) the anonymity of OFGs, which leads to more openness, and (c) feeling less inhibited and intimidated in an OFG. However, these results can merely be viewed as illustrative, as they were based on the answers of a small group of participants (N = 20). Moreover, the study included adult participants only, whereas OFGs are increasingly being used for younger age groups [e.g., [9, 10]. It would therefore be useful to investigate children’s and adolescents’ perceptions of OFGs as well.

The purpose of the present study was to fill a gap in the literature by providing insight into participants’ perceptions of OFGs in a large sample of children, adolescents and adults (N = 284, aged 8–72 years), who participated in an OFG. Participants’ preference for participation in an OFG or an FTF was studied, as well as their perceptions of the advantages of both methods. This allowed us to investigate whether the advantages of OFGs as perceived by their participants concur with the advantages of OFGs as a research method. We also investigated whether any differences exist between the perceptions of children, adolescents and adults. This information can be useful to adapt the design of OFGs to the preferences of their participants and thereby elicit the most optimal responses.

## Methods

A web-based application to conduct OFGs was developed as part of a previous study [8, 9]. This application was made available to other researchers, provided that we were allowed to send a short evaluative questionnaire to the participants of the OFGs.

Fifty OFGs were conducted between December 2005 and December 2013 as part of 19 separate studies (Table 1). All studies were executed in the Netherlands, except for one, which was executed in Switzerland. A total number of 420 persons participated in the OFGs, an average of 8 persons per OFG. The majority of participants were adults (71.2%) and female (60.2%) Forty-five percent of the OFG participants were patients/consumers, 43.3% were health care providers or other professionals, and 11.7% were parents (Table 2).

**Table 1:** **Characteristics of the 19 OFG studies**

Topic of the study	Year	N of OFGs	OFG participants	N participants OFG	N respondents questionnaire
Patients’, parents’ and survivors’ communication preferences in paediatric oncology	2005-2006	3	C, ado, adults	36	31
Evaluation of a website for patients with constitutional eczema	2007	2	Adults	27	23
Professional behaviour of medical students	2010	1	Adults	16	10
Consumers’ preferences regarding health care insurance options	2010	1	Adults	7	11
Health care providers’ opinions about adherence to guidelines in health care	2010	1	Adults	3	2
Health care providers’ opinions about case management for persons with dementia	2010-2011	13	Adults	100	34
Children’s and adolescents’ opinions about psychosocial burn care	2011	2	C, ado	19	15
Breast cancer patients’ experiences with hormonal therapy	2011	3	Adults	31	29
Parents’ opinions about shared medical appointments for children with diabetes	2011	2	Adults	8	4
Young patients’ perspectives on rehabilitation care	2011	2	C, ado	11	12
Children’s opinions about participation in an intervention for anxiety and depression	2011-2012	4	C, ado	44	32
Health care providers’ opinions about palliative care after a stroke	2012	1	Adults	17	12
Experts’ opinions about prevention in primary health care	2012	1	Adults	10	4
Satisfaction of primary health care professionals within collaborative partnerships	2012	2	Adults	5	5
Cancer survivors’ and physicians’ preferences regarding communication about fertility	2012	4	Adults	21	12
Adherence and self-management of children with asthma	2013	3	C, ado, adults	23	15
Preferences regarding information about medication of patients with rheumatism	2013	1	Adults	10	8
Adherence of adolescents with asthma	2013	2	Ado	14	12
Health care providers’ experiences with district nurse care	2013	2	Adults	18	13

c = children; ado = adolescents.

**Table 2:** **Characteristics of OFG participants and respondents of the questionnaire**

	OFG participants (N = 420)		Respondents of questionnaire N = 284	
	N	%	N	%
**Age in categories**				
Child (<12 years)	58	13.8	41	14.4
Adolescent (12–20 years)	63	15.0	52	18.3
Adult (>20 years)	299	71.2	158	55.6
Unknown	-	-	33	11.6
**Gender**				
Male	108	25.7	78	27.5
Female	253	60.2	185	65.1
Unknown	59	14.1	21	7.4
**Type of participant/respondent**				
Health care provider/other professional	182	43.3	84	29.6
Patient/consumer	189	45.0	162	57.0
Parent/guardian	49	11.7	38	13.4

The OFGs were conducted in an asynchronous form [6, 13], i.e. participants could read others’ comments and could respond at any time, not necessarily simultaneous with someone else’s participation. This allowed participants to respond from their home at any time convenient to them. The participants received individual login names and passwords, with which they could anonymously access the OFG website.

OFGs lasted 11 days on average, ranging from 5 to 30 days. In most OFGs, the first 5–7 days were used by the researchers to post one question or statement per day, whereas the remaining days were used to give participants the opportunity to react to questions of previous days and to the reactions of other participants. The researchers monitored the discussion and asked additional questions when necessary.

After the end of each OFG, the participants were asked to fill in an online questionnaire in which the method of OFGs was being evaluated. Topics were derived from the literature on the advantages and disadvantages of OFGs versus FTFs. The questionnaire started with a question regarding the preference for participation in an OFG or an FTF. Respondents were subsequently asked to rate on a predetermined list which advantages they perceived of participating in OFGs or FTFs, respectively. They could also fill in any additional advantages. Respondents who preferred to participate in an OFG were asked to indicate the advantages of OFGs, whereas respondents who expressed a preference for participation in an FTF were asked to indicate the perceived advantages of FTFs. Respondents who expressed no preference for either OFG or FTF were requested to rate the advantages of both methods.

The remaining questions addressed the duration of the OFGs, and whether or not to introduce all questions at the start of the OFG. Questions for children, adolescents and adults were comparable in content, but the wording was adapted to the age range of the respondents.

Invitations to fill in the questionnaire were also sent to persons who agreed to participate in the OFG but eventually did not post any reactions on the website (N = 150, data were not complete for three OFGs). A reminder was sent one week after the first request. A total number of 284 persons completed the questionnaire (of whom 14 did not post any reactions in the OFG). Characteristics of the respondents are reported in Table 2. All child and adolescent respondents were patients/consumers. Approximately half of the adult respondents (51.3%) were health care providers or other professionals, 33.5% were patients/consumers and 15.2% were parents or guardians (data not shown in Table 2).

Results may have been clustered within OFGs. For instance, some perceived advantages (e.g. the preference of participating anonymously) may have been more relevant in OFGs in which sensitive topics were discussed than in other OFGs. We tested whether results were clustered within OFGs by means of multilevel analyses. Since our analyses did not show significant effects of clusters, traditional chi square tests with p < 0.05 were used to test differences in perceived advantages of OFGs and FTFs between children, adolescents and adults. When significant chi squares were found, post-hoc tests with Bonferroni correction (p < 0.02) were used to investigate between which age groups the difference exists.

### Ethics

Respondents received information about the aim of our study in an information letter preceding their participation in the OFG, and again with the invitation to fill in the questionnaire, after the end of each OFG. The anonymity of the respondents was strictly ensured throughout the process of data collection and analysis. According to the Dutch ‘Medical Research Involving Human Subjects Act’ , ethical approval is not required for this kind of survey research, which does not involve any intervention.

## Results

When asked whether they preferred to participate in an OFG or an FTF, the majority of respondents (64.4%; N = 183) expressed a preference for participation in an OFG. A minority (23.6%; N = 67) expressed a preference for participation in an FTF, and 34 respondents (12.0%) had no preference for either OFG or FTF. There were no significant differences between the preference to participate in an OFG or an FTF between children, adolescents and adults (chi square test, p > 0.05).

The perceived advantages of participating in an OFG or an FTF are reported in Tables 3 and 4.

**Table 3:** **Advantages of OFGs according to child, adolescent and adult respondents (N = 217), in percentages**

	Child N = 26	Adolescent N = 42	Adult N = 123	Total N = 217	p
I am able to participate at a moment most convenient to me	30.8	90.5	95.9	86.2	0.00*
I can react from my own home and do not have to travel to participate	65.4	69.1	68.3	69.1	0.99
Responding via the Internet gives me more time to think about my answers	50.0	42.9	44.7	46.1	0.29
I am better at expressing my answers when I have to write them down	42.3	26.2	21.1	24.4	0.06
I prefer to give my answers anonymously	26.9	19.1	19.5	19.4	0.61
I find it easier to discuss personal issues through the Internet	23.1	23.8	11.4	15.7	0.02*
Other advantages**	11.5	4.8	4.1	4.6	0.27

Numbers of respondents with a preference for participation in an OFG or with no preference for OFG or FTF are reported. Because the age of 26 of these respondents was unknown, the total number of respondents exceeds the sum of the three subgroups.

*Differences between the age groups are statistically significant (chi-square test, p < 0.05).

**The most important other advantage of OFGs mentioned by respondents was that it is easier to stick with the original subject in OFGs than in FTFs (N = 3).

**Table 4:** **Advantages of FTFs according to child, adolescent and adult respondents (N = 101), in percentages**

	Child N = 21	Adolescent N = 17	Adult N = 51	Total N = 101	p
It is easier to have a discussion with the whole group when there is personal contact with others	4.8	17.7	78.4	48.5	0.00*
I like to see who I am talking to	52.4	70.6	39.2	45.5	0.07
I am better at expressing myself orally	47.6	17.7	29.4	29.7	0.13
I prefer to express my opinions in personal contact with others	23.8	35.3	45.1	33.7	0.23
I find it easier to discuss personal issues in direct contact with others	14.3	5.9	9.8	9.9	0.76
An FTF costs less time than a OFG that lasts several days	0.0	11.8	15.7	9.9	0.16
Other advantages**	4.8	11.8	15.7	13.9	0.44

Numbers of respondents with a preference for participation in an FTF or with no preference for OFG or FTF are reported. Because the age of 12 of these respondents was unknown, the total number of respondents exceeds the sum of the three subgroups.

*Differences between the age groups are statistically significant (chi-square test, p < 0.05).

**The most important other advantage of FTFs mentioned by respondent was that FTFs provide more opportunities for clarification and nuance than OFGs (N = 4).

### Perceived advantages of OFGs

Most respondents of the questionnaire valued the possibility to participate at a moment most convenient to them (86.2%) and to participate from their own home (69.1%). The possibility to respond anonymously in an OFG was mentioned as an advantage by merely 19.4% of the respondents. A minority of respondents (15.7%) reported that they found it easier to discuss personal issues via the Internet.

The most important additional advantage of OFGs spontaneously reported by respondents was that it is easier to stick to the original topic in OFGs than in FTFs (N = 3):

‘*On the Internet, it’s easier to stick to the subject or to ask directly what you want to know (…). This is not the case in a face-to-face discussion; if the topic does not appeal to you, you quickly get the feeling that you don’t belong there.’* (female respondent, 42 years old).

Another advantage mentioned was the absence of visual cues in an OFG (N = 1):

*‘In a face-to-face discussion, you can easily get distracted, and sometimes also influenced by the way someone looks, talks etc. This doesn’t bother you in an online discussion.’* (female respondent, 54 years old).

There were some significant differences between the advantages perceived by children, adolescents and adults. Post-hoc tests showed that adolescents and adults more often reported the possibility to participate at a moment most convenient to them as an advantage of an OFG than children did (90.5% and 95.9% versus 30.8%, p < 0.02). A considerable percentage of children and adolescents (23.1% and 23.8%) reported that they found it easier to discuss personal issues via the Internet, whereas only 11.4% of adults perceived this as an advantage of OFGs (p < 0.05). Pairwise comparisons with Bonferroni correction showed a significant difference between adolescents and adults on this item (p < 0.02).

### Perceived advantages of FTFs

The most important advantage of FTFs was respondents’ perception that it is easier to have a discussion with the whole group in an FTF (mentioned by 48.5% of respondents). This advantage was mentioned significantly more often by adults (78.4%) than by children and adolescents (4.8% and 17.7%, p < 0.02). About half of respondents (45.5%) perceived the possibility to see who they are talking to as an advantage of FTFs.

The most important spontaneously reported advantage of FTFs (i.e. not mentioned in the predetermined list of advantages in the questionnaire) was that FTFs provide more opportunities for clarification and nuance than OFGs (N = 4):

*‘You’re able to see whether someone understands what you’re saying and you’re able to clarify your answer when needed.’* (female respondent, 49 years old).

### Respondents’ opinions about OFGs

In the OFGs, a new question or statement was posted each day. Respondents were asked whether they found this method acceptable, or whether they would have preferred to see all questions at once. Most respondents (61.6%) preferred to see a new question each day, whereas 28.2% would have preferred to see all questions at the same time. These opinions did not differ significantly between the three age groups (chi square test, p > 0.05).

OFGs lasted 11 days on average, ranging from 5 to 30 days. The majority of respondents (64.8%) regarded the duration of the OFG as appropriate, 26.4% regarded the duration as too short, and 2.5% as too long. This opinion did not differ significantly between the three age groups (chi square tests, p > 0.05).

At the end of the questionnaire, respondents were asked whether they had any suggestions to improve the OFG application or any other remarks regarding the OFG. The remark mentioned most (N = 15 respondents) was that it is difficult to have a real discussion with the whole group:

*‘It was no group discussion, but answers to questions.’* (female respondent, 47 years old).

*‘There was hardly any discussion. This may have been caused by the way the questions were raised, which left us little room for discussion. It was more a situation of questions and answers than a discussion.’* (female respondent, age unknown).

Other important suggestions had to do with sending more frequent reminders (e.g. each time a new question or statement was posted on the website or each time another participant had posted a reaction; mentioned by 14 respondents), and posing clear and non-overlapping questions/statements that elicit a discussion (N = 12).

## Discussion

OFGs have been shown to be a feasible tool for collecting data from children and adolescents as well as adults [2, 5–9]. As found in previous research [8], most respondents valued the convenience of participating in an OFG at their own time and place most. Whereas the possibility to respond from their own home was valued equally by all age groups, the flexibility to respond at a moment most convenient to them was probably more relevant for the busy lives of adolescents and adults, since they reported this significantly more often as an advantage of OFGs than children did.

Previous research [8] has suggested that the anonymity of OFGs may cause participants to feel more comfortable to express their views. OFGs are therefore suggested to be particularly useful to discuss sensitive or personal issues [2, 13, 15, 16]. In our study, however, only a minority of respondents (19.4%) perceived the anonymity of OFGs as an advantage, and merely 15.7% of respondents found it easier to discuss personal issues via the Internet. This finding may be ascribed to the fact that a considerable number of studies included in this paper did not address particularly sensitive topics. The finding that adolescents significantly more often than adults reported the ease to discuss personal issues via the Internet as an advantage of OFGs may reflect their familiarity with the Internet.

Reid and Reid [16] found that participants’ reasons for preferring participation in an FTF rather than in an OFG were mainly related to the communication flow. Participants in that study found that FTFs provided a more fluid debate and that it was easier to follow the discussion in an FTF. Similarly, we found that the most important perceived advantage of FTFs was respondents’ perception that it was easier to have a discussion with the whole group in an FTF than in an OFG. This also corresponds with the remarks made by some OFG participants that the OFG in which they participated hardly had the character of a group discussion, but could merely be viewed as answers to questions. This underlines the importance of posing questions or statements that provoke discussion and the important role of the moderator in enabling a constructive discussion. Of course, these requirements do not only apply to OFGs, but to FTFs as well.

Apart from this, our study provided some additional suggestions for future researchers who want to organise an OFG. Firstly, most participants prefer a new question or statement to be posted each day. Secondly, sending reminders during the OFG, for instance each time a new question is posed or each time another participant has posted a reaction, will probably increase participants’ involvement and participation in the OFG.

### Limitations

Although invitations to complete the evaluative questionnaire were also sent to persons who agreed to participate in an OFG but eventually did not post any reactions (of whom 14 responded), respondents with a preference for participating in OFGs may have been more likely to fill in the questionnaire, thereby possibly overestimating the preference for OFGs. Moreover, advantages of OFGs and FTFs were investigated in OFG participants only, we did not include any FTF participants in our survey. Although this may have affected our results, a considerable number (N=67) of our OFG participants expressed a preference for participation in an FTF and could therefore provide valuable information about the advantages they perceive in FTFs. However, respondents’ opinions about FTFs may have been based on their perception of what participation in an FTF would be like, rather than on their actual experience with an FTF.

The OFGs from which we derived our data were conducted as part of 19 separate studies by other researchers. Respondents’ opinions about OFGs may have been influenced by the way in which the OFG was organised. Except for seven OFGs, in which the first author functioned as moderator, we did not have any influence on the questions or statements that were raised in the OFGs, nor did we have any insight into the dynamics and content of the OFGs that may have affected participants’ views.

Some of the advantages mentioned by our respondents (e.g. the convenience of participating in an OFG at their own time) particularly apply to asynchronous OFGs [cf. [18]. Future research should also focus on the perceived advantages of synchronous online focus groups, in which participants are online simultaneously and immediately react to each other’s responses.

## Conclusions

Our results show some strengths and weaknesses of OFGs and FTFs, which we believe can contribute to the debate regarding the merits of both methods. Participants’ perceptions of OFGs partly concur with the advantages of OFGs as a research method. Whereas respondents generally value the convenience of participating at their own time and place, the anonymity of OFGs and the increased ease to discuss personal issues were valued less by participants. A weakness of OFGs, or at least an aspect that needs considerable attention when organising an OFG, can be the lack of a fluid discussion, which is, according to our respondents, easier to achieve in an FTF.
